# Plasma von Willebrand Factor Is Elevated Hyperacutely After Mild Traumatic Brain Injury

**DOI:** 10.1089/neur.2023.0044

**Published:** 2023-10-11

**Authors:** Rachel Thomas, Cillian E. Lynch, Jeff Debad, Christopher Campbell, Onyinyechi Chidomere, Joseph Kilianski, Kan Ding, Christopher Madden, Danielle K. Sandsmark, Ramon Diaz-Arrastia, Joshua W. Gatson

**Affiliations:** ^1^ Department of Neurology, University of Pennsylvania Perelman School of Medicine, Philadelphia, Pennsylvania, USA.; ^2^ Meso Scale Diagnostics, LLC, Rockville, Maryland, USA.; ^3^ Department of Surgery, University of Texas Southwestern Medical Center, Dallas, Texas, USA.; ^4^ Department of Neurology, University of Texas Southwestern Medical Center, Dallas, Texas, USA.; ^5^ Department of Neurological Surgery, University of Texas Southwestern Medical Center, Dallas, Texas, USA.; ^6^ General Dynamics Information Technology, Falls Church, Virginia, USA.

**Keywords:** biomarker, trauma, traumatic brain injury (TBI), von Willebrand factor (vWF)

## Abstract

Each year in the United States, ∼2.7 million persons seek medical attention for traumatic brain injury (TBI), of which ∼85% are characterized as being mild brain injuries. Many different cell types in the brain are affected in these heterogeneous injuries, including neurons, glia, and the brain vasculature. Efforts to identify biomarkers that reflect the injury of these different cell types have been a focus of ongoing investigation. We hypothesized that von Willebrand factor (vWF) is a sensitive biomarker for acute traumatic vascular injury and correlates with symptom severity post-TBI. To address this, blood was collected from professional boxing athletes (*n* = 17) before and within 30 min after competition. Plasma levels of vWF and neuron-specific enolase were measured using the Meso Scale Discovery, LLC. (MSD) electrochemiluminescence array-based multi-plex format (MSD, Gaithersburg, MD). Additional symptom and outcome data from boxers and patients, such as the Rivermead symptom scores (Rivermead Post Concussion Symptoms Questionnaire [RPQ-3]), were collected. We found that, subsequent to boxing bouts, there was a 1.8-fold increase in vWF levels within 30 min of injury (*p* < 0.0009). Moreover, fold-change in vWF correlates moderately (*r* = 0.51; *p* = 0.03) with the number of head blows. We also found a positive correlation (*r* = 0.69; *p* = 0.002) between fold-change in vWF and self-reported post-concussive symptoms, measured by the RPQ-3. The receiver operating curve analysis of vWF plasma levels and RPQ-3 scoring yielded a sensitivity of 94.12% and a specificity of 76.5% with an area under the curve of 83% for boxers after a fight compared to the pre-bout baseline. This study suggests that vWF is a potential blood biomarker measurable in the hyperacute period after blunt mild TBI. This biomarker may prove to be useful in diagnosing and monitoring traumatic vascular injury.

## Introduction

Traumatic brain injury (TBI) is a common cause of morbidity and mortality, both globally and domestically.^1^ Annually, TBI results in approximately 2.7 million emergency department (ED) visits within the United States, the majority of which are classified as mild, based on Glasgow Coma Scale (GCS) score and a brief period of unconsciousness or confusion.^2,3^ Despite being labeled as mild, many of these injuries can have long-term neuropsychiatric sequelae, including chronic traumatic encephalopathy (CTE), post-traumatic seizure, and chronic headache.

Blood biomarkers have the potential to improve clinical management by identifying injury mechanisms of secondary brain injury^4–12^ and thus allowing for targeted therapeutic interventions. Over the past decade, numerous studies have reported the diagnostic accuracies of blood levels of neuroglial biomarkers, such as glial fibrillary acidic protein (GFAP), ubiquitin carboxyl-terminal hydrolase 1 (UCH-L1), neuron-specific enolase (NSE), and S100 calcium-binding protein B (S100B), for identifying TBI patients likely to have acute traumatic intracranial abnormalities on brain computerized tomography (CT) imaging,^13,14^ and the assessment of S100B has been incorporated into Scandinavian neurotrauma guidelines.^15^ These biomarker tests are promising triage tools to aid clinical decision-making regarding the need for neuroimaging. The U.S. Food and Drug Administration has cleared the use of GFAP and UCH-L1 for aid in the evaluation of TBI, given that a low concentration of these proteins in the blood is associated with the absence of acute traumatic abnormalities on a brain CT.^16^ However, most studies have measured blood samples collected between 3 and 12 h post-injury,^14,17^ which is reasonable in the ED, but not early enough to be clinically informative at the scene of an injury, sidelines of a sporting event, or military combat settings.

In addition to the neuronal and glial injury described above, traumatic impacts to the brain also damage the cerebral vascular endothelium and surrounding glial cells that together make up the neurovascular unit (NVU).^18^ In response to trauma, endothelial cells increase the production of von Willebrand factor (vWF) to initiate a plethora of vascular repair mechanisms.^19^ This large glycoprotein is produced by endothelial cells and platelets, and is released from endothelial cells post-injury to promote platelet aggregation.^20–22^ In addition to facilitating platelet aggregation, vWF also activates leukocytes, playing a role in inflammation and immunothrombosis, a process by which activated leukocytes interact with platelets and coagulation factors, leading to intravascular clot formation and microthrombosis.^23^

Previous studies have found that plasma vWF levels are elevated in response to numerous physiological and physical insults.^24–28^ In several published reports, vWF has been demonstrated to be involved in proinflammatory processes after traumatic subarachnoid hemorrhage complicated by cerebral vasospasm.^29–37^ In blast and TBI victims, acute cerebrospinal fluid levels of vWF were elevated as early as 6 h post-injury and were found to correlate with more significant injury on CT scan^38^ and poor outcomes.^19,38–40^ Therefore, if measured hyperacutely, vWF may represent an early biomarker of NVU injury and assist with both immediate post-TBI triaging and initial clinical management.

To better assess vWF in a hyperacute time frame useful for pre-hospital assessment, we aimed to measure plasma vWF levels after mild TBI (mTBI) in the hyperacute period (within 1 h) as a potential biomarker of microvascular injury. Professional boxing frequently results in competitors experiencing repeated mTBI, allowing within-subject comparison of vWF factors levels before and immediately after a fight. Here, we hypothesized that boxers would have increased plasma vWF levels post-bout and that such levels would correlate with the number of head blows and self-reported post-concussion symptoms.

## Methods

### Enrollment procedures

We established relationships with approximately 20 trainers and 100 professional boxers at 40 boxing facilities across North Texas to perform the prospective MAMBA (Mild and Moderate TBI Biomarker) study. This study was approved before initiation by the institutional review board at UT Southwestern Medical Center (Dallas, TX). In brief, boxers between the ages of 18 and 35 years that competed in at least three 3-min rounds were eligible for the MAMBA study. Eligible boxers were approached at their training facilities within 2 weeks before a scheduled bout. Informed consent was obtained and baseline demographic and TBI history collected. Study subjects were assessed during and at the completion of their bout by the ringside physician, boxing trainer, or a member of the study team to assess the number of subconcussive or concussive blows. During the competition, three independent study team members counted the number of blows to the head, allowing an assessment of the average number of blows per participant. The Rivermead Post Concussion Symptoms Questionnaire (RPQ-3) was administered within 1 h of the bout to assess early post-concussive symptoms, including headaches, nausea and/or vomiting, and dizziness. The boxer was enrolled into the study if they had at least 25 blows to the head and an RPQ-3 score ≥2. Control subjects were community-dwelling adults recruited in the Dallas/Fort Worth area and had no history of a brain injury by self-reporting.

### Blood collection/storage

Blood was collected once at baseline (4 days before a bout) and within 15–30 min after completion of the bout and scoring >2 on the RPQ-3. In brief, at each time point, 8 mL of blood was collected and centrifuged at 4°C for 10 min at 1500 relative centrifugal force. Plasma was collected and aliquoted into 1-mL tubes and immediately frozen at −80°C. In control subjects, blood was collected at a single time point.

### Detection of von Willebrand factor and neuron-specific enolase

Samples were provided to Meso Scale Discovery, LLC (MSD, Gaithersburg, MD) for testing of vWF and NSE plasma levels using electrochemiluminescence immunoassays in an array-based multi-plex format.^41^ Briefly, capture antibody for vWF or NSE was printed on an electrode array spot within the wells of 96-well MULTI-ARRAY^®^ plates. Detection antibody was conjugated with an electrochemiluminescent SULFO-TAG™ label. Diluents containing various blockers were used to reduce background and heterophilic antibody interference. Plates were read on a SECTOR^®^ Imager 6000 reader.

Samples and calibrator dilutions were assayed in duplicate. Each plate also contained a control sample made from a plasma pool and eight dilutions of a recombinant vWF or NSE calibrator. Calibrator data were fitted with a four-parameter logistic curve fit and used to quantitate control and sample vWF and NSE concentrations. Detection limits were 0.3 μg/mL for vWF and 17 pg/mL for NSE. The calibration curve was also used to estimate the upper limit of the immunoassay's linear range. The final reported concentration of vWF and NSE was the mean of two replicates.

### Statistical analysis

Data were analyzed using the Student's *t*-test, and groups were considered to be significantly different if *p* values were <0.05 (SPSS, Inc., Chicago, IL). Simple linear regressions determined relationships between vWF, RPQ symptom scoring, and number of blows to the head. Correlations among groups were performed using Spearman's rank order. Receiver operating curve (ROC) analysis was used to find the plasma vWF cutoff that would identify symptomatic brain injury as determined by RPQ-3 score ≥2 within the boxers. The two groups included in this analysis were the baseline and post-bout vWF levels for the boxers. Area under the curve (AUC), sensitivity, specificity, positive predictive value, negative predictive value, likelihood ratio, and accuracy were measured. IBM SPSS Statistics (V20, 2011; IBM Corp., Armonk, NY) and GraphPad software (GraphPad Software Inc., San Diego, CA) were used to analyze these data. The data are presented as a scatter plot/line graph (GraphPad Software) depicting the mean ± standard deviation (SD).

## Results

### Subject demographics

The majority of subjects were male (67% of controls, 87.5% of boxers). The mean age of the controls and boxers was 28 and 23 years, respectively. The boxers were professional fighters with an average boxing experience of 5.8 years. The mean number of blows to the head during the bout was 48.7 (SD = 23). All of the boxers had 25 or more blows to the head during competition. Additionally, all of the boxers had an RPQ-3 score of ≥2 (Table 1).

**Table 1.** Subject Demographics

	Control (*n* = 21)	Boxer (*n* = 17)
Age (years), mean (SD)	28 (6.2)	23 (4.8)
Sex, male, *n* (%)	14 (67)	15 (87.5)
Boxing experience (years), mean (SD)	N/A	5.8 (3.6)
No. of previous concussions, mean (SD)	N/A	4.8 (2.2)
No. of blows to the head, mean (SD)	N/A	48.7 (27.5)
Symptom score, mean (SD)	N/A	2.7 (0.6)
Glasgow Coma Scale (GCS) score (SD)	N/A	N/A

SD, standard deviation; GCS, Glasgow Coma Scale; N/A, not applicable.

### Plasma von Willebrand factor and neuron-specific enolase levels are elevated in concussed professional boxers

**FIG. 1.**
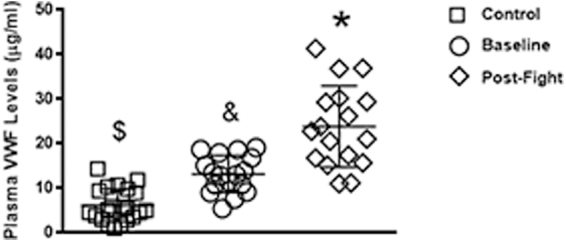
Plasma vWF levels are elevated in concussed professional boxers. A significant increase in plasma vWF was observed in boxers post-fight (⋄) compared to non-injured controls (□; ^$^*p* = 0.003) and pre-fight baseline levels (○; *p = 0.0009). No significant difference was observed between the non-injured control and baseline groups (^&^*p* = 0.623). vWF, von Willebrand factor.

**FIG. 2.**
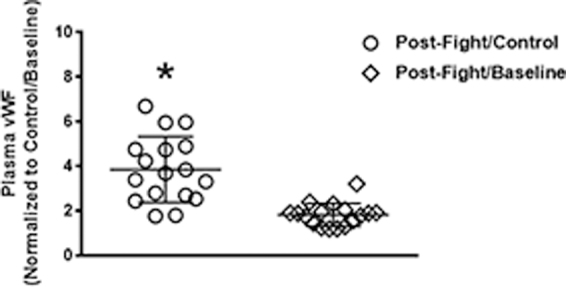
Differences in plasma vWF were more evident in professional boxers compared to non-injured control subjects. A 1.8- and 3.8-fold increase was observed in concussed athletes compared to non-injured control (○) and baseline groups (⋄), respectively. vWF, von Willebrand factor.

**FIG. 3.**
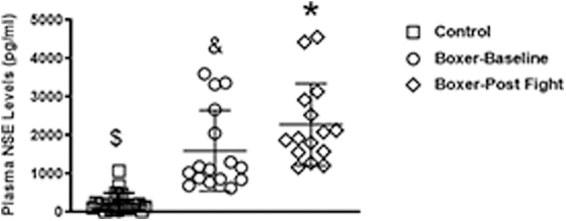
Plasma levels of a well-established acute brain biomarker, NSE, was significantly elevated in concussed professional boxers. When compared to non-injured controls (□; *p = 0.0001) and boxers at the baseline pre-fight time point (○; *p = 0.05), concussed professional boxers (⋄) had a significant increase in plasma NSE. A significant difference (^$^*p* = 0.001) in plasma NSE was also observed between non-injured controls and boxers at the baseline pre-fight time point. NSE, neuron-specific enolase.

vWF was quantitated in plasma collected before competition (baseline) and at ∼30 min after competition in 17 boxers. We found that compared to non-boxer/athlete controls (∼6.16 ± 3.69 μg/mL; *N* = 21), boxers had elevated pre-fight (baseline) vWF levels (∼13.15 ± 4.14 μg/mL), which was not significant (*p* < 0.62). At 30 min post-fight, a significant increase (*p* < 0.0009) in plasma vWF (23.09 ± 9.79 μg/mL) was observed between boxer baseline and post-fight levels (Fig. 1). vWF plasma levels in boxers after the bout were increased by 3.9-fold (range, 1.8–6.7) normalized to control subjects and 1.8-fold (range, 1.2–3.2) compared to baseline levels measured in the boxers (Fig. 2). In order to confirm that repeated blows to the head results in neural injury, we measured NSE, a well-characterized marker for neural injury. Here, we found that boxers had significantly higher plasma NSE compared to controls (*p* < 0.0001) and baseline levels (*p* < 0.05; Fig. 3).

### Plasma levels of von Willebrand factor correlate with outcomes after mild traumatic brain injury

**FIG. 4.**
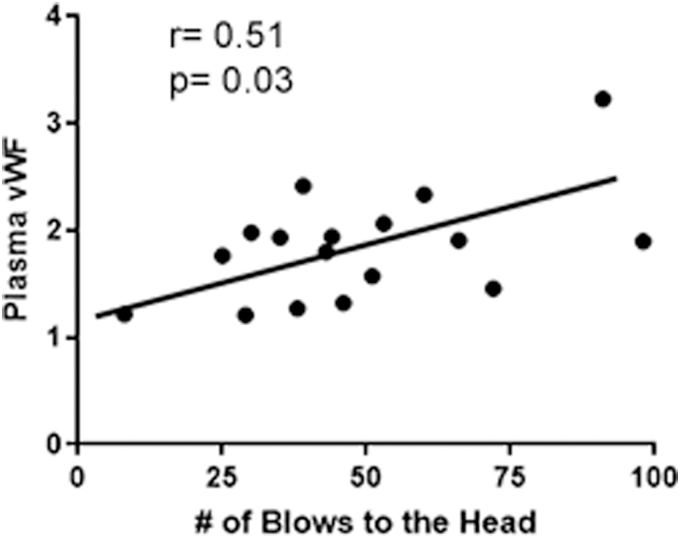
In concussed athletes, number of blows to the head correlated with the plasma levels of vWF. Linear regression demonstrating that fold-change in plasma vWF correlates moderately (*r* = 0.51; *p* = 0.03) with head blows. vWF, von Willebrand factor.

**FIG. 5.**
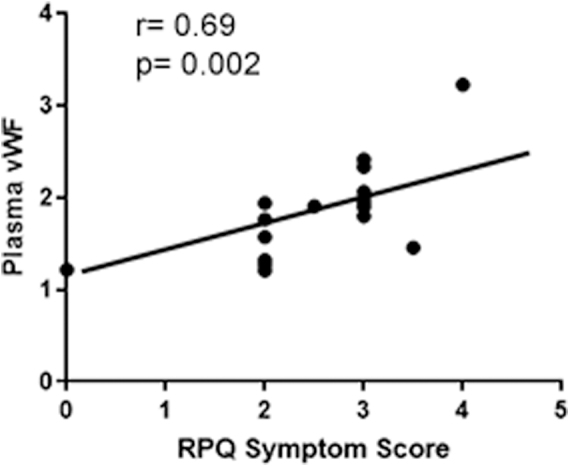
Symptom scoring in concussed boxers correlates with fold-change in plasma levels of vWF. Linear regression demonstrating the change in plasma vWF from baseline to post-bout correlates moderately with the post-bout RPQ-3 score (*r* = 0.69; *p* = 0.002). RPQ-3, Rivermead Post Concussive Symptoms Questionnaire; vWF, von Willebrand factor.

Using a simple linear regression, we found that fold-change in plasma vWF correlates moderately (*r* = 0.51; *p* = 0.03) with the number of blows to the head at 30 min after concussion in boxers (Fig. 4). The change in plasma vWF from baseline to post-bout was also correlated with the post-bout RPQ-3 score (*r* = 0.69; *p* = 0.002; Fig. 5).

### von Willebrand factor receiver operating curve

**FIG. 6.**
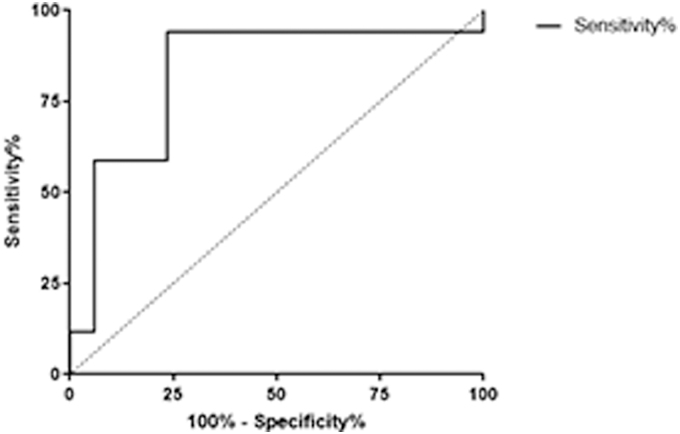
Detection of plasma vWF levels is a sensitive/specific marker in symptomatic concussed boxers. Using an ROC analysis comparing pre- and post-injury plasma vWF levels of professional boxers, the AUC was found to be 0.83. Using a cut-off value of a 1.9-fold increase of plasma vWF, a sensitivity of 94.12% and specificity of 76.47% was achieved (*p* = 0.001). AUC, area under the curve; ROC, receiver operating characteristic; vWF, von Willebrand factor.

To determine the change in plasma vWF that identifies which subjects may be experiencing heightened secondary brain injury, we conducted an ROC analysis. We compared post-injury plasma vWF levels of professional boxers to pre-bout plasma vWF levels. For the professional boxers, the AUC was found to be 0.83. Using the cut-off value of a 1.9-fold increase of plasma vWF (post-injury normalized to baseline), a sensitivity of 94.12% and specificity of 76.47% was achieved (*p* = 0.001; Fig. 6).

## Discussion

We found that vWF is significantly elevated in the blood in a similar manner as NSE as early as 30 min post-injury. To further support our findings, we conducted an ROC analysis and elucidated that vWF is both a sensitive and specific biomarker in concussed athletes. The case presented here indicates that vWF levels increase quickly after repetitive TBIs.

Here, we enrolled professional boxers with mTBI and found that vWF is significantly elevated in the blood within 30 min post-TBI (Figs. 1–3). This suggests that injury of the vascular endothelium may be occurring. Repetitive mTBI, despite its frequent initial paucity of clinical sequelae, contributes to long-term functional disability and poses a substantial risk for later development of dementia/CTE.^42,43^ Traumatic neurovascular injury has been shown to result in blood–brain barrier (BBB) compromise, cerebral blood flow dysregulation, and microthrombosis, which have been linked to poor outcomes, and its potential role in neurodegeneration is an area of active research.^44,45^ In evaluating TBI, the dearth of established clinical biomarkers presents a challenge toward the accurate assessment of the extent of injury and the development of effective therapies. A sensitive and reliable biomarker of vascular injury may be useful for selecting patients for clinical trials of vascular-targeted therapies.^44^

Another compelling aspect of using vWF as an early marker of vascular injury is the reliable detection of this biomarker at very early time points, as demonstrated here within 30 min of sports-related concussion. This is an important property of vWF given that identification of injuries in pre-hospital settings, such as accident sites, sidelines of sporting events, and military combat settings, will allow for appropriate triage and referral for emergency care and more likely reduce long-term cognitive deficits and even death. Moreover, the use of plasma, as in this study, rather than serum biomarkers offers the advantage of using non-clotted blood, therefore negating the time requirement for the specimen to coagulate before processing. Given that vWF is a critical component of the clotting complex, it is primarily found in plasma rather than serum, thus making it more amenable to use in point-of-care applications.

Because vWF is not specific to the brain, one pitfall of using vWF as a lone biomarker is the lack of specificity at identifying whether a person has an intracranial injury. To overcome this, the addition of vWF on a multi-biomarker panel of established brain and inflammatory biomarkers may provide additional information regarding neurovascular injuries. This approach is thought to increase the predictive value of vWF especially if an increase in brain biomarkers correlates with the levels of vWF. Interestingly, these data suggest that repetitive mTBI may disrupt BBB integrity, as has been suggested by past studies in sports-related mTBI.^46^ Our view is that vWF may be useful alongside other well-characterized brain/inflammatory biomarkers such as NSE,^47^ GFAP,^14^ UCH-L1,^14^ and neurofilament-light chain.^48^

In conclusion, our data demonstrate that plasma vWF increases after mild, repetitive TBI and changes in vWF correlate with symptom severity immediately subsequent to impacts. Future investigation to determine the utility of vWF on a brain biomarker panel to assist in clinical decisions for neuroimaging and/or various interventional paradigms is warranted. Importantly, in a more heterogeneous patient population, it may help determine the degree of vascular injury, delineating specific TBI endophenotypes and potentially selecting subjects for trials of therapies targeting microvascular injury.

## Data Availability

Eligible boxers were approached at their training facilities within 2 weeks before a scheduled bout. Informed consent was obtained at that time to obtain baseline demographic and TBI history. Study subjects were assessed during and at the completion of their bout by the ringside physician, boxing trainer, or a member of the study team to assess the number of subconcussive or concussive blows. During the competition, three independent study team members counted the number of blows to the head and recorded symptoms, allowing assessment of the average number of blows per participant. The boxer was enrolled into the study if they had an RPQ-3 score ≥2 and at least 25 blows to the head. Consent was then obtained for study participation as well as to publish their data.

## Abbreviations Used

AUC: area under the curve
BBB: blood–brain barrier
CT: computed tomography
CTE: chronic traumatic encephalopathy
ED: emergency department
GCS: Glasgow Coma Scale
GFAP: glial fibrillary acidic protein
mTBI: mild TBI
NSE: neuron-specific enolase
ROC: receiver operating characteristic
RPQ-3: Rivermead Post Concussion Symptoms Questionnaire
S100B: S100 calcium-binding protein B
SD: standard deviation
TBI: traumatic brain injury
UCH-L1: ubiquitin carboxyl-terminal hydrolase 1
vWF: von Willebrand factor
